# Draft Genome Sequence of Corynebacterium sanguinis Strain Marseille-P8776

**DOI:** 10.1128/mra.00008-22

**Published:** 2022-05-04

**Authors:** Mudra Khare, Dhiraj Sinha, Rita Zgheib, Amael Fadlane, Didier Raoult, Pierre-Edouard Fournier

**Affiliations:** a Aix Marseille University, Institut de Recherche pour le Développement (IRD), Service de Santé des Armées, Assistance Publique-Hôpitaux de Marseille, UMR Vecteurs Infections Tropicales et Méditerranéennes (VITROME), Institut Hospitalo-Universitaire Méditerranée Infection, Marseille, France; b Institut Méditerranée-Infection, Marseille, France; c Aix-Marseille Université, Institut de Recherche pour le Développement (IRD), UMR Microbes Evolution Phylogeny and Infections (MEPHI), Institut Hospitalo-Universitaire Méditerranée-Infection, Marseille, France; University of Maryland School of Medicine

## Abstract

In 2018, Corynebacterium sanguinis strain Marseille-P8776 was isolated from the blood of a 64-year-old woman suffering from breast cancer who had undergone chemotherapy and radiotherapy. Following whole-genome sequencing, the chromosome of strain Marseille-P8776 was 2,613,836 bp long with a G+C content of 64.92%, 2,568 protein-coding genes, and 64 RNA genes.

## ANNOUNCEMENT

The genus *Corynebacterium* was created by Lehmann and Neumann in 1896, but the main features of this genus were first described by Collins and Cummins in 1986 (1). Members of the genus are distributed in various places such as soil, water, plants, and food products, and some species are commensals of mucosa and skin from humans and animals (2). Currently, the genus *Corynebacterium* is composed of 136 species (https://lpsn.dsmz.de/genus/corynebacterium) (3). In 2018, we isolated strain Marseille-P8776 from a blood sample taken from a 64-year-old woman suffering from breast cancer and being treated with radiotherapy and chemotherapy at La Timone Hospital (Marseille, France).

Bacterial growth was obtained following culture on 5% sheep blood-enriched Columbia agar (bioMérieux, Marcy l’Etoile, France) in an aerobic atmosphere at 37°C for 24 h. Genomic DNA (gDNA) from strain Marseille-P8776 was quantified using a Qubit assay (Life Technologies, Carlsbad, CA, USA) to 0.2 ng/μL. Next, the gDNA was sequenced using a MiSeq sequencer with the paired-end strategy and barcoded using the Nextera XT DNA sample prep kit (Illumina Inc., San Diego, CA, USA).

The genomic DNA was diluted to obtain 1 ng as input. In the tagmentation step, the DNA was fragmented and tagged. Then, using limited-cycle PCR amplification (12 cycles), the tag adapters were completed, and dual-index barcodes were introduced. After purification on AMPure XP beads (Beckman Coulter Inc., Fullerton, CA, USA), the libraries were normalized on specific beads according to the Nextera XT protocol (Illumina). The pooled single-strand library was loaded onto the reagent cartridge and then onto the instrument along with the flow cell. Automated cluster generation and paired-end sequencing with dual index reads were performed in a single 39-h run in 2 × 250-bp format.

Total information of 3.9 Gb was obtained from a 491 K/mm^2^ cluster density with a cluster passing quality control filters of 93.50%. Within this run, the index representation of strain Marseille-P8776 was 3.83%. The 9,478,951 paired-end reads were filtered according to the read quality using FastQC version 0.11.8 with default parameters (https://www.bioinformatics.babraham.ac.uk/projects/fastqc/). The genome assembly was performed as previously described (4) using the SPAdes version 3.11.1 software (5) that includes GapCloser and PEResolver for gap and overlap removal (6). The “careful” option was used to minimize the number of mismatches and short indels. Default parameters were applied for k values, i.e., k-mer values of 21, 33, 55, 77, 99, and 127. The genome sequence was annotated using the NCBI Prokaryotic Genome Annotation Pipeline (PGAP) version 4.12 (7) The predicted protein sequences were compared to the Clusters of Orthologous Groups (COGs) database using BLASTP (E value, 1e-03; coverage, 0.7; and identity, 30%). Strain Marseille-P8776 had a genome size of 2,613,836 bp assembled into one scaffold with a G+C content of 64.92%. Of 2,847 predicted genes, 2,691 were protein-coding genes and 63 RNAs (4 rRNAs, 55 tRNAs, and 4 noncoding RNAs [ncRNAs]). A total of 1,937 genes (73.59%) were assigned a putative function by COGs, and 695 genes (26.40%) were annotated as hypothetical proteins.

Using ABRicate software (8), the genomic sequences were screened against the CARD (Comprehensive Antibiotic Resistance Database) (9) for resistance genes and the VFDB (Virulence Factors Database) (10) for virulence factors, revealing that strain Marseille-P8776 harbored 6 resistance genes and 7 virulence genes.

### Data availability.

The draft genome sequence of *C. sanguinis* strain Marseille-P8776 has been deposited at GenBank under accession number NZ_JACEOR000000000.1 and the raw reads under SRA accession number SRR17477869 (BioProject accession number PRJNA645897 and BioSample accession number SAMN15518506).
